# Chlorogenic Acid and Its Microbial Metabolites Exert Anti-Proliferative Effects, S-Phase Cell-Cycle Arrest and Apoptosis in Human Colon Cancer Caco-2 Cells

**DOI:** 10.3390/ijms19030723

**Published:** 2018-03-03

**Authors:** Shima Sadeghi Ekbatan, Xiu-Qing Li, Mohammad Ghorbani, Behnam Azadi, Stan Kubow

**Affiliations:** 1 School of Human Nutrition, McGill University, 21111 Lakeshore, Ste Anne de Bellevue, Montreal, QC H9X 3V9, Canada; shima.sadeghi@mail.mcgill.ca (S.S.E.); behnam.azadi@mcgill.ca (B.A.); 2 Fredericton Research and Development Center, Agriculture and Agri-Food Canada, Fredericton, NB E3B 4Z7, Canada; Xiu-Qing.Li@agr.gc.ca; 3 The Rosalind & Morris Goodman Cancer Research Center, McGill University, 1160 Pine Ave West, Montreal, QC H3A 1A3, Canada; mohammad.ghorbani@mail.mcgill.ca

**Keywords:** Caco-2 cells, chlorogenic acid, caffeic acid, 3-phenylpropionic acid, benzoic acid, cell cycle, caspase-3, apoptosis

## Abstract

Chlorogenic acid (CGA) decreases colon cancer-cell proliferation but the combined anti-cancer effects of CGA with its major colonic microbial metabolites, caffeic acid (CA), 3-phenylpropionic acid (3-PPA) and benzoic acid (BA), needs elucidation as they occur together in colonic digesta. Caco-2 cancer cells were treated for 24 h with the four compounds individually (50–1000 µM) and as an equimolar ratio (1:1:1:1; MIX). The effective concentration to decrease cell proliferation by 50% (EC_50_) was lower for MIX (431 ± 51.84 µM) and CA (460 ± 21.88) versus CGA (758 ± 19.09 µM). The EC_50_ for cytotoxicity measured by lactate dehydrogenase release in MIX (527 ± 75.34 µM) showed more potency than CA (740 ± 38.68 µM). Cell proliferation was decreased by 3-PPA and BA at 1000 µM with no cytotoxicity. Cell-cycle arrest was induced at the S-phase by CA (100 µM), MIX (100 µM), CGA (250 µM) and 3-PPA (500 µM) with activation of caspase-3 by CGA, CA, MIX (500 and 1000 µM). Mitochondrial DNA content was reduced by 3-PPA (1000 µM). The anti-cancer effects occurred at markedly lower concentrations of each compound within MIX than when provided singly, indicating that they function together to enhance anti-colon cancer activities.

## 1. Introduction

Colorectal cancer is among the most common causes of cancer deaths worldwide [1]. There is a large body of evidence supporting the role of fruit and vegetable consumption in reducing the risk of different types of cancer, including colorectal cancer [1]. A variety of nutrients and phytochemicals present in fruits and vegetables have been targeted as potential anti-cancer factors. Among the proposed chemopreventative food components, polyphenols have been consistently indicated to play a major anti-cancer role [1]. A diet rich in polyphenols has been indicated to contribute towards the health of the gastrointestinal tract, which is the site exposed to high concentrations of those compounds [2]. In that regard, various animal models and in vitro cell-culture studies representing different stages of colonic cancer have provided supportive evidence for the anti-carcinogenic effects of some polyphenols [1]. Polyphenols can exert anti-cancer properties via a variety of mechanisms, which are not yet fully understood. These mechanisms include induction of cell-cycle arrest and modulation of various oncogenic signaling cascades that affect cell proliferation and apoptosis [3]. Polyphenols could also exert anti-cancer activities by damaging mitochondrial DNA or via mitochondrial DNA depletion, as such effects could lead to autophagy and the induction of apoptosis [4].

Chlorogenic acid (CGA) is the major dietary polyphenol in many populations due to their high consumption of coffee, a rich source of CGA [5]. An inverse association between coffee intake and colon cancer has been reported in epidemiological studies, which has been related to a higher intake of CGA [5]. CGA, an ester of caffeic acid (CA) with quinic acid, is partly absorbed in the upper GI tract in its intact form; however, most of ingested CGA (approximately 70%) is cleaved in the lower GI by gut microflora resulting in the release of free CA and additional microbial metabolites with varying biological effects such as 3-phenypropionic acid (3-PPA) and benzoic acid (BA) [6,7]. Despite promising human and animal data indicating the anti-cancer effects of CGA [8], the mechanisms of action have not been studied extensively. Several investigations have treated various cultured cancer cell types with CGA and CA as single compounds at physiological and supraphysiological concentrations [1,3,8]. In such studies, these compounds showed anti-proliferative effects that were related to the stimulation of the expression of several apoptosis-associated genes [9] and to cell-cycle arrest [1,3].

The aim of this study was to evaluate the anti-cancer effects of CGA and its metabolites (CA, 3-PPA and BA) on the human colonic Caco-2 cancer cell line, which is a well-utilized colorectal cancer model. The effects of an equimolar combination of the above four compounds (MIX) was tested as gut epithelial cells are exposed to both parent compounds and their microbial metabolites. For that assessment, measurements included cell proliferation, cytotoxicity, cell-cycle events, caspase-3 activation and mitochondrial DNA content. To our knowledge, this is the first study to relate the anti-cancer effects of CGA in combination with its microbial metabolites in colonic tumor cells, which could be physiologically relevant based on their concurrent presence in gut digesta.

## 2. Results

### 2.1. Cell Proliferation and Cytotoxicity

**Figure 1:**
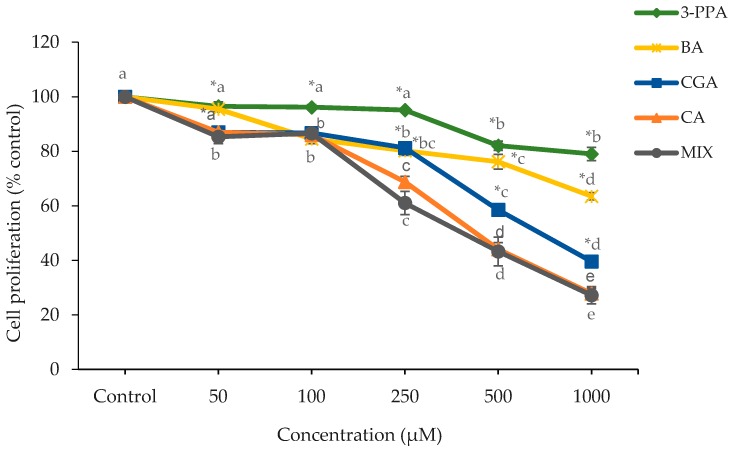
Effect of treatment with different doses of CGA, CA, 3-PPA, BA and MIX for 24 h on Caco-2 cell proliferation as measured by the MTT assay. Data are represented as mean ± standard error (SE). Statistical analysis was performed via two-way analysis of variance (ANOVA) using treatment and dose as factors. Doses within the same treatment not sharing common letters are significantly different (*p* < 0.05). The symbol * represents a significant difference (*p* < 0.05) of CA and MIX as compared to CGA, 3-PPA and BA at a specific dose. CGA = chlorogenic acid; CA = caffeic acid; 3-PPA = 3-phenylpropionic acid; BA = benzoic acid; MIX = equimolar mixture of the four tested compounds.

**Figure 2:**
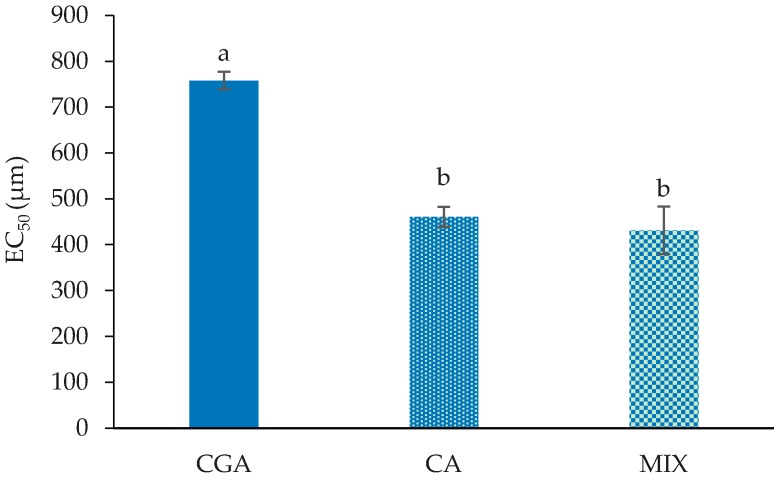
The concentrations of CGA, CA and MIX that decrease cell viability by 50% (EC_50_). Data are represented as mean ± SE. Statistical analysis was performed via one-way ANOVA. Bars not sharing the same letters are significantly different (*p* < 0.05) from each other. CGA = chlorogenic acid; CA = caffeic acid; MIX = equimolar mixture of the four tested compounds.

Caco-2 cells have been used extensively as an in vitro model of the exposure of cancer cells to bioactive dietary components and chemotherapeutic agents. The influence of CGA and CGA metabolites on the growth of Caco-2 cells as measured by the MTT (3-(4,5-dimethylthiazolyl-2)-2,5-diphenyltetrazolium bromide) assay at 24 h is shown in Figure 1. The significant decrease (*p* < 0.05) in cell proliferation by CGA, CA and MIX treatment started at the lowest tested concentration (50 µM) (Figure 1). However, at the lower concentrations (50 and 100 µM), they only exerted slight (<20%) anti-proliferative effects. In terms of CGA, a substantial decrease (42.5%) in cell proliferation was noted at 500 µM (*p* < 0.05) with a further reduction (60.4%) seen at 1000 µM (*p* < 0.05). In contrast to CGA, the CA- and MIX-treated cells showed significant effects (*p* < 0.05) on proliferation starting at a lower concentration of 250 µM, with decreases of 31.2% and 38.94%, respectively. The CA and MIX treatments showed significantly lower cell proliferation (*p* < 0.05) at 250, 500 and 1000 µM relative to CGA. Treatment with CA and MIX showed dose-dependent reductions (*p* < 0.05) at 500 µM (55.9% and 56.7%) and 1000 µM (72.2% and 72.8%). Cell proliferation was affected by BA only at higher concentrations with a slight decrease in cell proliferation starting at 100 µM (*p* < 0.05) and further (*p* < 0.05) dose-related decreases at 250, 500 and 1000 µM. Relative to BA, significantly greater reductions (*p* < 0.05) in proliferation were seen at 50, 500 and 1000 µM for CGA and at 50, 250, 500 and 1000 µM for CA and MIX. Cell proliferation was affected only to a small extent (*p* < 0.05) for 3-PPA at 500 and 1000 µM. CGA, CA and MIX had significantly greater decreases (*p* < 0.05) in cell proliferation at all concentrations than 3-PPA. BA-treated cells also showed significantly greater decreases (*p* < 0.05) in proliferation than 3-PPA at 100, 250 and 1000 µM. Due to their inability to decrease cell proliferation by 50%, an EC_50_ was not obtained for 3-PPA and BA. Both 3-PPA and BA, however, appear to have contributed to the anti-proliferative effect in MIX as the concentration to decrease cell proliferation by 50% (effective concentration; EC_50_) for MIX was 431 ± 51.84 µM. The EC_50_ for CGA was significantly higher (*p* < 0.05) than for MIX and CA (Figure 2), which reflected a lower antiproliferative potential for CGA. In that regard, the EC_50_ for MIX had a combined concentration of the two major anti-proliferative compounds of CGA and CA (215.5 µM) that was markedly lower than the EC_50_ concentrations of the two compounds individually, 758 ± 19.09 µM and 460 ± 21.88 µM, respectively.

**Figure 3:**
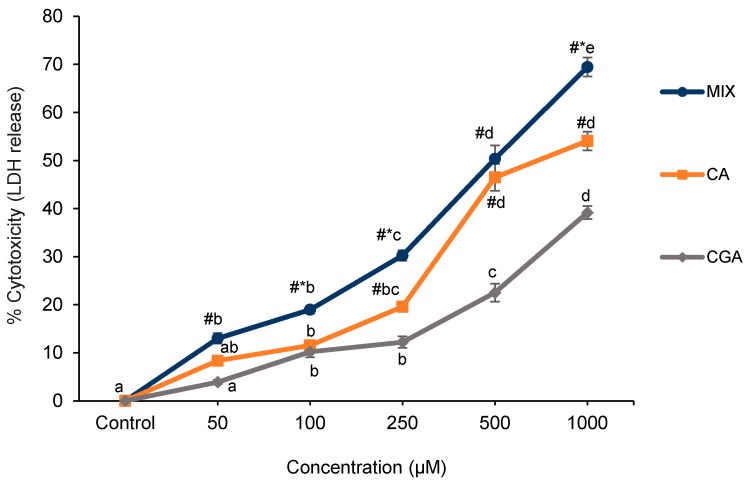
Effect of treatment with different doses of CGA, CA and MIX for 24 h on Caco-2 cell cytotoxicity as evaluated by the LDH assay. Data are represented as mean ± SE. Statistical analysis was performed via two-way ANOVA using treatment and dose as factors. Doses within the same treatment not sharing common letters are significantly different (*p* < 0.05). The symbol # represents a significant difference (*p* < 0.05) between CGA and either of the two other treatments at a specific dose. The symbol * represents a significant difference (*p* < 0.05) between MIX and CA at a specific dose. CGA = chlorogenic acid; CA = caffeic acid; MIX = equimolar mixture of the four tested compounds; LDH = lactate dehydrogenase.

The lactate dehydrogenase (LDH) assay is complementary to MTT as it describes the release of intracellular LDH into the culture medium, which indicates that cell-membrane damage resulted in irreversible cell death [10]. The CGA, CA and MIX treatments caused significant concentration-dependent increases in LDH release compared to control (*p* < 0.05) although only slight increases in cytotoxicity were noted at the lower concentration range of 50–250 µM (Figure 3). Treatment with CA and MIX showed dose-dependent increases in LDH release (*p* < 0.05) at 500 µM (46.5% and 50.4%) and 1000 µM (54% and 69.5%). The CA and MIX treatments exerted significantly greater cytotoxicity (*p* < 0.05) as compared to CGA at 250 µM (12.2%), 500 µM (22.5%) and 1000 µM (39.2%). Both BA and 3-PPA showed no significant effect on LDH release. The MIX combination treatment (250 µM each for CGA, CA, BA, 3-PPA) showed stronger cytotoxicity (69.5%) (*p* < 0.05) relative to CGA (39.2%) and CA (54.1%) at the highest dose of 1000 µM. The obtained EC_50_ for MIX (527 ± 75.34 µM) was significantly lower (*p* < 0.05) and thus showed a higher cytotoxic potential than the EC_50_ value for CA (740 ± 38.68 µM). CGA treatment did not reach values to obtain an EC_50_ within the tested concentration range. The results thus show a combined action on cytotoxicity of CGA and its microbial-generated metabolites in MIX as these effects occurred at lower concentrations of the single compounds in MIX than when those agents were tested separately.

### 2.2. Cell-Cycle Analysis

To understand the possible mechanisms for cell death, the cell cycle was measured, as the cytotoxicity of polyphenols has been related to induction of apoptosis and cell-cycle arrest by various mechanisms [1,11,12]. The regulatory checkpoints of the G_1_/S and G_2_/M phase that repair damaged DNA are defective in cancer cells, which allows their unregulated proliferation of the cells. Consequently, cell-cycle disruption has been a focus of anti-cancer therapies to inhibit cancer-cell growth [13]. To determine whether the growth inhibitory effects of the test compounds resulted from growth arrest, the cell-cycle response of Caco-2 cells was examined by flow cytometry.

**Figure 4:**
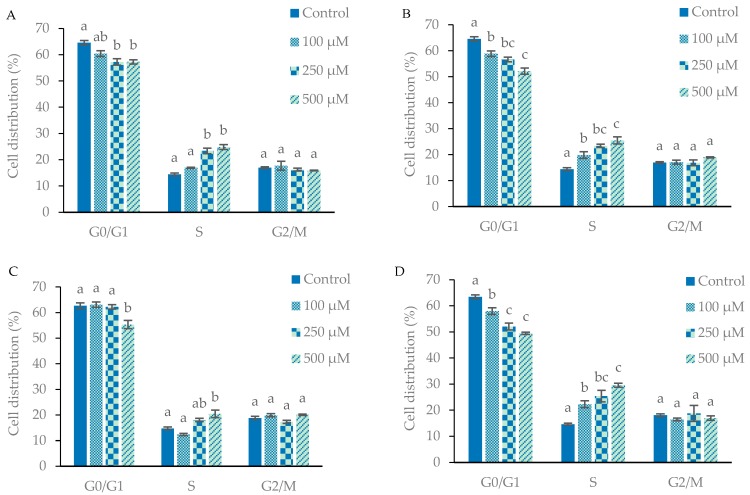
Effects of chlorogenic acid (**A**); caffeic acid (**B**); 3-phenylpropionic acid (**C**) and MIX (**D**) treatments for 24 h on Caco-2 cell-cycle distribution as determined by flow cytometry. Data are represented as mean ± SE. Bars not sharing the same letters within each cell cycle are significantly different (*p* < 0.05) from each other.

Evaluation of the disruption of the distribution of cells in the G_0_/G_1_, S and G2/M phases of the cell cycle took place following incubation with CGA, CA, 3-PPA and MIX at doses of 100, 250 and 500 µM for 24 h (Figure 4). For CGA, CA, 3-PPA and MIX, a dose-related significant reduction (*p* < 0.05) of cells in the G_0_/G_1_ phase was noted with no significant increase in cells in the G_2_/M phase. BA treatment did not show any effect on cell-cycle distribution. CA and MIX significantly lowered (*p* < 0.05) the percentage of G_0_/G_1_ cells following the 100 µM treatment with a corresponding increase of cells in the S-phase. On the other hand, CGA caused significant reduction (*p* < 0.05) in the G_0_/G_1_ and increase in S-phases only at the higher concentration of 250 µM. Further significant reductions (*p* < 0.05) of the G_0_/G_1_ phase and increases in S-phase were noted following incubation of MIX and CA at 250 and 500 µM, respectively. Treatment with 3-PPA for 24 h caused significant reduction (*p* < 0.05) in the G_0_/G_1_ and increase in S-phases at 500 µM. The cell-cycle analyses thus signified that CGA, CA, 3-PPA and MIX caused a cell-cycle arrest at the S-phases in a dose-dependent manner. The effects of MIX on the arrest of the S-phases at 100 and 250 µM occurred with a combined content of CGA and CA (50 and 125 µM, respectively) that was markedly lower than seen with the effects of the single compounds CGA (250 and 500 µM) and CA (100 and 500 µM). The cell cycle findings were therefore in line with the combined effects of CGA and its metabolites on cell proliferation and cytotoxicity exerted by MIX on Caco-2 cells.

### 2.3. Apoptosis

**Figure 5:**
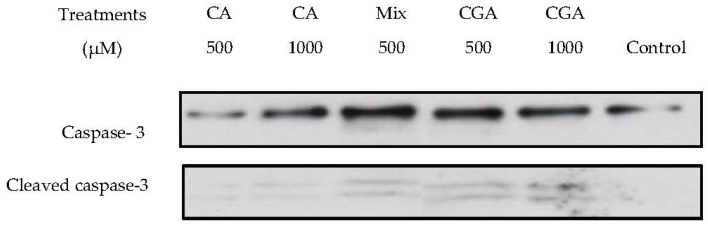
Effect of CGA, CA and MIX treatment for 24 h on cleaved and uncleaved caspase-3 levels as evaluated by Western blotting. CGA = chlorogenic acid; CA = caffeic acid; MIX = equimolar mixture of the four tested compounds.

Activation of apoptosis in cancer cells is one of the pathways induced by chemopreventative or chemotherapeutic compounds including pharmaceuticals [14] as well as naturally occurring compounds such as phenolics [15]. Activation of at least one caspase is essential to induce cellular apoptosis as these enzymes annul the effect of protective factors towards cellular integrity such as the DNA-repair enzyme poly(ADP-ribose) polymerase (PARP). To confirm the induction of apoptosis, the next step was to analyze the expression of caspase-3, which is one of the major enzymes involved in the initiation of apoptosis. Activation of caspase-3 is a confirmed target of apoptosis induction as the application of active staining of caspase-3 and cleaved caspase-3 is a validated marker of apoptosis for cancer cells [16]. Therefore, we investigated the activation of caspase-3 as a possible apoptotic pathway induced by CGA, CA and MIX treatments in Caco-2 cells. To further confirm the activity, the expression level of the caspase-3 protein was studied. Detection of cleaved caspase-3 bands was performed and this indicates complete activation of caspase-3 by cleavage of pro-caspase-3. Figure 5 shows expression of caspase-3 after exposure to 500 and 1000 µM of CGA and CA and 500 µM of the MIX treatment. Relative to controls, only the 1000 µM dose of CA was associated with an increase in expression in caspase-3, whereas a similar level of expression was noted for CGA at 500 and 1000 µM. An elevated activity of caspase-3 was supported by an observed increase in the expression of cleaved caspase-3 bands. The MIX treatment containing a combined content of 250 µM for CGA and CA appeared to demonstrate a similar degree of expression in caspase-3 and cleaved caspase-3 to the 1000 µM concentrations of CGA and CA, which is supportive of effects exerted by the combination of compounds in MIX.

### 2.4. Mitochondrial DNA Content

**Figure 6:**
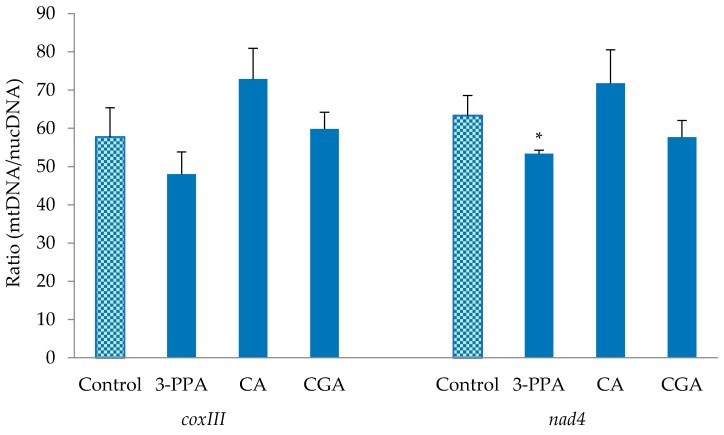
Effect of 24 h treatment with 1000 µM CGA and its metabolites on Caco-2 cells on the mtDNA content (ratio of mtDNA/nucDNA) as measured by droplet digital polymerase chain reaction (PCR). Data are represented as mean ± SE. CGA = chlorogenic acid; CA = caffeic acid; 3-PPA = 3-phenylpropionic acid. *: significant from the control (*p* < 0.05).

The effects of CGA and its metabolites (CA, 3-PPA) on mitochondrial DNA content, in terms of the ratio of mitochondrial DNA (mtDNA) to nuclear DNA (nucDNA), was assessed since phytochemicals such as curcumin [4,17] and certain chemotherapeutic drugs cause damage to mtDNA, which can be a mechanism for induction of apoptosis in cancer cells [17]. Two mitochondrial genes (*NAD* and *Cox*) were tested. For the *NAD* gene, treatment with 3-PPA significantly reduced (*p* < 0.05) the mtDNA/nucDNA ratio compared with the control (Figure 6). A similar tendency obtained for 3-PPA was seen with the mitochondrial *Cox* gene (Figure 6). The results are indicative of involvement of depletion of mitochondrial DNA content in the induction of apoptosis by 3-PPA in Caco-2 cells.

## 3. Discussion

The results of the current study demonstrate for the first time that treatment with a combination of CGA and its microbial metabolites CA, BA and 3-PPA exerts greater effects than the single compounds on cytotoxicity and the inhibition of human colon cancer-cell proliferation, involving cell-cycle arrest and apoptosis. These findings were achieved using concentrations of CGA and its microbial breakdown products detected in human fecal content [18,19] and human digesta [7,20]. Following ingestion of 1000 mg CGA (2.8 mmol), about 67% was recovered in ileal fluid of ileostomy subjects [20] which is higher than the highest tested concentration in the present study. Likewise, 71% of 385 µM ingested CGA (≈275 µM) was recovered in ileal effluent [7] which is in the middle of the range of tested concentrations of CGA used in the present work. The values of CA seen in human fecal water of 52–126 µM [18] correspond to the lower range of the tested concentrations. Previous human fecal studies have shown a wide range of 3-PPA concentrations of 1747–2136 µM [19], 45–417 µM [21] and 165–440 µM [18] that corresponds to the range of the studied concentrations. Similarly, BA has shown a large range of values in human fecal water of 2060–4625 µM [18] and 51–134 µM [18].

Although such compounds are poorly absorbed [22], their relatively high concentrations in the gut lumen could exert functional anti-colon cancer effects. There are a limited number of studies regarding the anti-cancer effects of CGA and CA on colon cancer cell lines, including Caco-2 cells. Similar to the present findings, CGA treatment of human HT29 colon cancer cells showed anti-proliferative effects after 72 h at concentrations of 289.2 µM [23] and 500 µM [24]. The greater anti-proliferative and cytotoxic potency of CA as compared to CGA is also in agreement with previous work that showed more effective anti-proliferative effects on human HT29 colon cancer cells at a lower dose (EC_50_: 235.1 µM after 48 h) compared to CGA (EC_50_: 289 µM after 72 h) [23]. In another study, CA decreased cell viability in HT29 cells at an earlier time point than CGA (24 h vs. 96 h) [25]. The chemical structural of the polyphenols determine their anti-cancer efficacy. It has been shown that the aromatic ring and hydroxyl groups are key functional element required for the anti-cancer activity of polyphenols [3]. The esterification of the carboxyl group of CA with quinic acid could affect the efficacy of the biological activity exerted by CGA [26].

The present investigation extends these previous findings by demonstrating that the combination of CGA with CA, BA and 3-PPA in the MIX treatment led to a net increase in bioactivity in terms of the inhibition of cell proliferation, cytotoxicity, and cell-cycle arrest as compared to those compounds tested singly. The antiproliferative potential of the MIX combination showed a EC_50_ value that contained concentrations of CGA (107.7 µM) and CA (107.7 µM) that were notably lower than the EC_50_ concentrations of the two compounds individually, 758 and 460 µM, respectively. Similarly, the evaluation of the cytotoxic effect of the compounds showed a higher EC_50_ cytotoxic index for MIX than the individual compounds. The greater antiproliferative and cytotoxic effects associated with MIX are presumably associated with the combined presence of CGA and its breakdown products. It is noteworthy that despite minimal effects of either BA or 3-PPA alone on Caco-2 cells, their presence in MIX contributed towards an additive anti-cancer effect in concert with CGA and CA. Additive or synergistic antiproliferative effects on Caco-2 cells have been observed from the combination of polyphenols in extracts of cranberries [27] and pomegranates [28] as compared to the isolated polyphenols identified in those extracts. The benefits of additive or synergistic effects between polyphenols has been indicated as a potentially important aspect towards cancer prevention and treatment [29]. Such additive or synergistic actions can be due to the combination of complementary antiproliferative mechanisms induced by the different polyphenol compounds such as modulation of cell-cycle regulators like *p*53, or inhibition of molecular pathways involving mitogen-activated protein kinase, NF-κB and activator protein 1 [3].

Based on the antiproliferative effects induced by CGA and the CGA metabolites, cell-cycle progression was studied. The progression of the cell cycle is regulated by cyclins, cyclin-dependent-kinases (CDKs), CDK inhibitors and check-point kinases that regulate cell proliferation at the G_1_/S and G_2_/M cell-cycle checkpoints [13]. Dysregulated expression of these proteins can lead to tumorigenesis, and so modulating the cell cycle is a target pathway for chemoprevention [13]. Anti-cancer compounds can arrest the cell cycle at the G_0_/G_1_, S or G_2_/M phase to stimulate apoptotic events. The significant accumulation of cells in the S-phase with a concomitant decrease in G_0_/G_1_ indicated that Caco-2 cells treated with MIX, CGA, CA and 3-PPA had inhibited cell proliferation due to the blockage of the cell cycle at the S-phase. Overall, these findings indicate that these compounds at subtoxic concentrations can inhibit cell proliferation and the progression of the S-phase in the cell cycle. CGA and CA have been shown to induce cell-cycle arrest in other cell lines, including human sporadic colon-cancer cell lines (HCT116 and SW480) and human acute promyelocytic leukemia HL60 cells [11,12]. Previous studies in Caco-2 cells have also shown inhibition of cell proliferation involving S-phase arrest by polyphenol parent compounds including ferulic acid and *p*-coumaric acid [1] and resveratrol [30].

The antiproliferative activity associated from the Caco-2 cell treatments could be mediated by the induction of apoptosis, which can occur through different molecular pathways including modulation of the activity of the caspase family. Among the caspase family, the proapoptic protein caspase-3 plays an important role towards inducing apoptotic events received from both intrinsic and extrinsic apoptotic pathways [15]. Western blot experiments were undertaken to elucidate the involvement of caspase-3 as an apoptotic pathway in Caco-2 cells treated with CGA, CA and MIX. The presence of cleaved caspase-3 in the cells treated with CGA, CA and MIX demonstrated the capability of those compounds to cleave caspase-3. The findings showed that caspase-3 was activated in a dose-dependent manner by CGA. Increased caspase-3 activation from the combined compounds present in the MIX treatment was also suggested. CGA has been shown to activate caspase-3 through various pathways including production of reactive oxygen species and via activation of caspase-8 [31,32]. Caspase-3 activation is a confirmatory marker for apoptosis [16]. Previous studies have shown CGA-induced apoptosis in leukemia HL-60 cells [11] and that CA caused apoptosis and inhibited cancer-cell proliferation in HT-1080 human fibrosarcoma cell line [33] and human leukemia cells [34]. Apart from caspase-3, further studies are needed to provide insight regarding the other signaling pathways that could be involved in the apoptotic effects of CGA and its microbial breakdown compounds. Molecular targets could include apoptotic regulating proteins such as cyclin-dependent-kinases, NF-κB, Bcl-2, caspase-9 and PARP. Previous studies have indicated that polyphenols can affect cell cycles via different molecular pathways depending on the cancer-cell type and the chemical structure of the polyphenol [1,3].

Apoptosis induction in Caco-2 cells was independent of mtDNA content alterations for CGA, CA and BA in this study, which indicates that mtDNA was not involved in the mechanisms of action for the apoptotic events involving those compounds. Interestingly, maintaining an adequate balance in mtDNA content during the induction of apoptosis has been proposed as a promising avenue for cancer therapy as both decreased or increased levels of mtDNA content have been associated with resistance to anti-cancer drugs [35]. On the other hand, 3-PPA treatment was associated with reduction of mtDNA, which can be a mechanism for the apoptosis seen with this metabolite. Resveratrol-mediated depletion of mtDNA content in breast- and colon-cancer cells has been linked with autophagy as well as induction of caspase activation and apoptosis [36]. Curcumin treatment was also shown to involve mtDNA reduction as an apoptosis trigger in hepatoma HepG2 cells [17].

In summary, the combination of CGA and its microbial metabolites was shown to exert enhanced anti-cancer effects in Caco-2 cells at subtoxic levels that involve inhibition of cell proliferation reflected by a lengthening of the S-phase and apoptotic cell death. The study findings also demonstrate that involvement of mtDNA in apoptosis can vary according to the nature of the microbial metabolite as 3-PPA was the only metabolite associated with alteration of mtDNA, within the power of the tested biological replicates. These results support the contention that the combination of polyphenol metabolites formed during digestive processes can function together to increase anti-colon cancer effects. As such, these findings provide new avenues for in vitro and in vivo investigations regarding the anti-cancer roles of polyphenol-derived microbial compounds, which could lead to a better understanding of the mechanisms of action of dietary polyphenols in cancer chemoprevention.

## 4. Material and Methods

### 4.1. Cell Culture

Human colon cancer Caco-2 cells (adenocarcinoma) were obtained from the American Tissue Type Collection (ATCC, Burlington, ON, Canada) and cultured according to the company’s procedures as explained briefly below. The Caco-2 cells were cultured in minimum essential medium Eagle (MEM) supplemented with 10% fetal bovine serum (FBS), 1% nonessential amino acids and 0.1% penicillin-streptomycin. Cells were incubated at 37 °C with 5% CO_2_ and 90% humidity and were monitored daily. The cells were subcultured at 80% confluence with 0.25% trypsin EDTA solution for 5–10 min and were seeded in a new flask or seeded onto appropriate plates for different experiments. Cells were treated with different concentrations (50, 100, 250, 500 and 1000 µM) of CGA, CA, 3-PPA and BA and an equimolar mixture (MIX) of the four compounds (CGA + CA + 3-PPA + BA; 1:1:1:1, respectively) for 24 h. The stock solution of the tested compounds was prepared in DMSO and the final percentage of the DMSO in the cell culture media during the treatment was less than 0.1%. The highest dose of the test compounds of 1000 µM correspond to their maximal concentrations in digesta following the consumption of CGA or CA-rich foods diluted in an intestinal volume of 600 mL [18,37].

### 4.2. Analysis of Cell Proliferation

Cell proliferation was assessed using the MTT colorimetric assay. The MTT assay is based on the reduction of yellow tetrazolium MTT to purple formazan by the action of mitochondrial dehydrogenase in viable cells. To assess the effects of CGA and its metabolites on cell viability, a dose-response study using concentrations of 50, 100, 250, 500 and 1000 μM of the tested compounds was performed. Briefly, after each treatment the supernatant was collected and the MTT solution was dissolved in phenol red free medium, added to the cells and incubated for 3 h. The supernatant was then removed and the blue formazan crystals were dissolved in HCl-isopropanol and the absorbance was measured at 570 nm.

### 4.3. Cell Cytotoxicity Assay

The LDH assay is a colorimetric membrane integrity assay for the quantification of cytotoxicity based on the release of cytoplasmic LDH into culture media from damaged cells. The cells were treated with the tested compounds (50, 100, 250, 500, 1000 μM) for 24 h, supernatant was collected and LDH release into media was measured using a cytotoxicity detection kit (LDH; Roche, Mississauga, ON, Canada) according to the manufacturer’s protocol.

### 4.4. Cell Cycle by Flow Cytometry Analysis

Cells were prepared for cell cycle analysis according to a modification of a previously described method [38]. Briefly, cells were seeded onto 6-well plates, and after reaching 80% confluency, treated with the test compounds for 24 h. Cells were then washed with phosphate-buffered saline (PBS), trypsinized, and 1 × 10^6^ cells mL^−1^ were collected into 5 mL tubes. Cells were centrifuged for 5 min at 200× *g*, the supernatant was removed and cells were fixed with 1 mL of 70% ethanol and stored at −20 °C, where they were kept until DNA staining. For DNA staining, samples were centrifuged and washed with PBS twice and centrifuged at 400× *g* for 5 min. Then, 1 mL of DNA staining buffer including 0.25 g sodium citrate, 0.75 mL Triton X-100 (1%), 0.005 g ribonuclease A, which were all purchased from Sigma-Aldrich (Sigma-Aldrich, Oakville, ON, Canada); 0.025 g propidium iodide (PI) (Fisher Scientific, Ottawa, ON, Canada) and 250 mL distilled water were added to the cell pellet. Immediately before acquisition, samples were filtered with 70 µm micron filters (Fisher Scientific, Ottawa, ON, Canada) to avoid any clogging. Cells were then analyzed for cell-cycle distribution by BD FACSCalibur flow cytometer (Becton Dickinson, Mississauga, ON, Canada). The PI fluorescence was collected as FL2 at 585 nm using CellQuest software (Becton Dickinson, San Jose, CA, USA). For each sample, 10,000 events were acquired. The analyses of cell-cycle distribution were performed on duplicate samples of three independent experiments using CellQuest software.

### 4.5. Western Blot Analysis of Caspase-3 Expression

Following 24 h treatment with the test compounds, the supernatant was removed and cells were washed twice with cold PBS. Thereafter, cells were lysed with radioimmunoprecipitation assay (RIPA) buffer (Fisher Scientific, Ottawa, ON, Canada) and the lysates were collected by scraping with a cold plastic cell scraper. The cell suspension was transferred into a centrifuge tube and shaken for 15 min at 4 °C to lyse the cells. The lysate was centrifuged at 14,000× *g* in a precooled centrifuge for 15 min. The supernatant containing the cytoplasmic proteins was transferred to a new tube. The protein concentration was determined using the Coomassie (Bradford) protein assay. A 10 µg amount of protein was resolved on to 12% sodium dodecyl sulfate-polyacrylamide gel electrophoresis and transferred onto a nitrocellulose membrane using the wet-Western-blotting system. After blocking with 5% milk, the membrane was incubated at 4 °C overnight under shaking with the appropriate antibodies. The caspase-3 and cleaved caspase-3 bands were detected by caspase-3 (8G10) rabbit monoclonal antibody (#9665, Cell Signaling Technology, Beverly, MA, USA).

### 4.6. Mitochondrial DNA Content

To assess mitochondrial DNA content, cells were seeded onto 6-well plates and treated with 1000 µM of the test compounds for 24 h. The supernatant was collected and the cells were washed twice with cold PBS. Cells were scraped into 2 mL cold PBS using a rubber policeman and transferred to a centrifuge tube on ice. Cells were recovered by centrifugation at 1500× *g* for 10 min at 4 °C. The cell pellet was used for total genomic DNA extraction using the Qiagen Blood & Cell Culture DNA Mini Kit (Qiagen, Toronto, ON, Canada) according to the manufacturer’s protocol. DNA quality and quantity were assessed at 260 and 280 nm using a NanoDrop 1000 spectrophotometer (Thermo Fisher Scientific, Wilmington, DE, USA). Droplet Digital PCR (ddPCR) was conducted using QX200 Droplet Digital PCR System (Bio-Rad, Hercules, CA, USA) following the application guide. The PCR conditions and cycles were 95 °C for 5 min, 40 cycles of 95 °C for 30 s, 55 °C for 30 s, and 72 °C for 45 s, then 4 °C for 5 min and 90 °C for 5 min. Two mitochondrial DNA regions are used, one is from the *coxIII* gene (U35430.1HSU35430, *Homo sapiens* cytochrome c oxidase subunit III gene), and one is from the *nad4* gene (AY063363.1, *Homo sapiens* NADH dehydrogenase subunit 4 gene). For the mitochondrial *coxIII* gene, the forward primer was the Primer 6031 (our lab code) 5′-TCTCAGCCCTCCTAATGACCTC-3′ and revise primer was primer 6032 5′-CCTTGGTATGTGCTTTCTCGTG-3′, for mitochondrial DNA gene, forward primer was Primer 6029) 5′-GCCCTCGTAGTAACAGCCATTC-3′, and reverse primer was Primer 6029 5′-TGTGAGTGCGTTCGTAGTTTGA-3′. The mitochondrial *coxIII* and *nad*4 genes were designed in this study using Primer 3 (www.realtimeprimers.com) in this study. For the human nuclear genome, the forward primer (Primer 6057) was 5′-GACAGTCAGCCGCATCTTCT-3′ and reverse primer (Primer 6058) was 5′-TTAAAAGCAGCCCTGGTGAC-3′ [39]. For each treatment-gene combination, there were four biological replications and two ddPCR detection channels. The detail of the design of the primers and the ddPCR protocol will be published elsewhere.

### 4.7. Statistical Analysis

All data are expressed as mean ± standard error (SE). For cell-cycle analysis, one-way analysis of variance (ANOVA) was used to compare group means followed by Tukey’s post-hoc test after checking for outliers and verification of normality of distribution using the Shapiro–Wilk test. The MTT and LDH data were analyzed by two-way ANOVA using treatment and concentration as main factors followed by Tukey’s post-hoc test for multiple comparisons. Statistical significance was set at *p* < 0.05 and all statistical analyses were performed using SPSS 22.0. and SigmaPlot v. 13 (Systat Software Inc., San Jose, CA, USA). The data analyses were performed on duplicate samples of three independent experiments except for the DNA analysis that was carried out on four biological repeats.
